# Assessment of Heavy Metal Concentrations with Fractionation Method in Sediments and Waters of the Badovci Lake (Kosovo)

**DOI:** 10.1155/2020/3098594

**Published:** 2020-02-18

**Authors:** Avni Malsiu, Ilir Shehu, Trajče Stafilov, Fatmir Faiku

**Affiliations:** ^1^Department of Chemistry, Faculty of Mathematical and Natural Sciences, University of Prishtina, 10000 Prishtina, Kosovo; ^2^Institue of Chemistry, Faculty of Science, Ss. Cyril and Methodius University, P.O. Box 162, 1001 Skopje, North Macedonia

## Abstract

The concentrations of thirteen metals (Al, As, Ba, Cd, Co, Cr, Cu, Fe, Mn, Ni, Pb, V, and Zn) were analyzed in waters and sediments of the Badovci Lake. The total metal concentrations in the water followed the descending order: Fe > Al > Mn > Cu > Ba > Zn > As > Ni > Pb > V > Co > Cd > Cr, and the total metal content in the sediments also followed the descending order: Fe > Al > Mn > Ni > Cr > Pb > Ba > Zn > V > Cu > As > Co > Cd. According to EC 98/83, Al, Fe, and Mn at some sampling sites exceeded safety limits for drinking water, whereas other elements were at acceptable levels. The total content of Cr, Cu, Ni, Pb, and V in the sediments exceeded the target values of the New Dutch List. Using pollution indicators such as the contamination factor (CF) and geoaccumulation index (I_geo_), most of the samples were unpolluted to moderately polluted by Cu, Cr, Pb, V, and Ni. The values of the pollution load index (PLI) were more than one (>1), indicating progressive deterioration of the sediment quality. The enrichment factor (EF) for all the studied metals suggests their enrichments in sediments of the Badovci Lake. Most of the elements were found in the residual fraction strongly bonded to the crystalline component. Pb, Mn, and Cu were bound in the organic and exchangeable components. The extent of pollution by heavy metals in sediments of the Badovci Lake implies that the environmental condition is relatively stable, and attention should be paid to metals bonded in the extractable and organic phases. It is recommended to periodically monitor water and sediment quality.

## 1. Introduction

The quality of sediments influences the water quality in the aquatic environment. Pollution of the aquatic environment by heavy metals has acquired increasing attention due to endurance in the sediment, toxicity, and organic accumulation that can influence human health and ecosystems [1]. According to the study in [2], of all chemical pollutants, heavy metals present special ecological, biological, and health significance. Bottom sediments in all aquatic environments are reasonable and fact-finding sources of and information on processes and mechanisms occurring in aquatic ecosystems [3]. The behavior of the metals in natural waters is determined by the water chemistry and sediment composition [2]. Geological weathering, soil erosion, airborne dust, atmospheric transportation-precipitation, and anthropogenic activities including fertilizer leaching, sewage discharge, industrial wastewater, and urban construction are factors that enable heavy metals to enter the lakes [4]. Hydrological cycles, physical-chemical processes, and complex spatiotemporal variation enable remobilization of heavy metals from sediments into the water [5]. Responsibility for transportation of heavy metals, essential elements, and pollutants in the aquatic ecosystem might be from sediments [1]. Through the point sources (e.g., industrial, municipal and domestic wastes, and agricultural fertilizers and pesticides) and diffuse sources (e.g., surface runoff, soil erosion, and atmospheric deposition), heavy metals enter the aquatic ecosystems [6, 7]. The total heavy metal content in the sediment, related to toxicity, mobility, and bioavailability, do not provide complete information, however, can be considered as pollution indicators [8]. By analyzing mobility, bioavailability, and chemical nature of elements, additional information can be obtained for heavy metals in the sediments. Using sequential extraction procedures with various extracting reagents, one can get information about sources and metal binding forms [1, 9–11]. The region where the Badovci Lake lies is known for the industrial activity such as the Artana and Kishnica mines, so the impact of human activity is inevitable. The Kishnica mine is located very close to the lake, and its activity releases many residues, which under the influence of the weather conditions affect the whole region near it, so it is rightly called a hot spot in Kosovo [12]. In the research done in [13], waters of the Gracanka River, which is part of the stream from the Badovci Lake, in some sampling sites have a high concentration of metals. The water analysis of the Badovci Lake done in [14] shows the presence of some toxic metals such as Pb, Cd, and Zn which are related to the geological structure of the soil and mining activities. Therefore, based on these data, our research is focused on analyzing the sediments and waters of this lake. The main objectives of this research are to assess the water and sediment quality status by estimating the levels of heavy metals in the water and sediments through fractionation methods and pollution indicators.

## 2. Materials and Methods

### 2.1. Description of the Study Area

**Figure 1:**
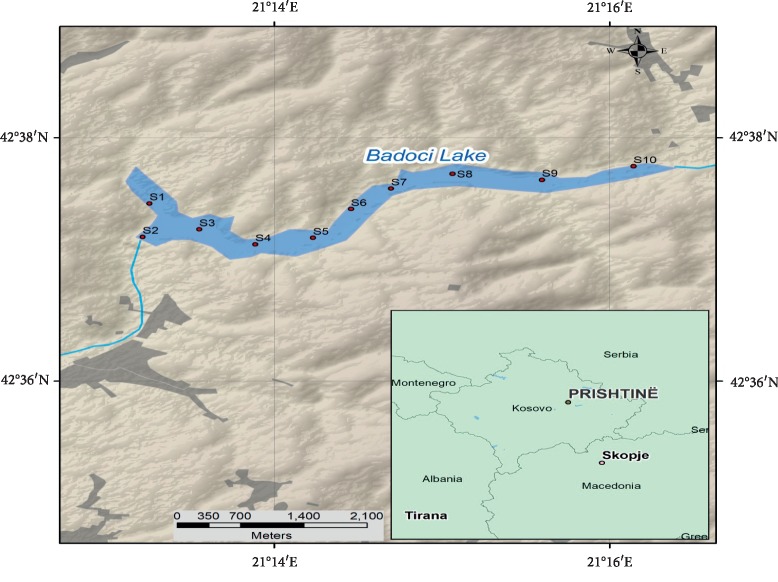
Map of the artificial lakes in Kosovo and study area, Badovci Lake.

Recent work is focused on water and sediment quality of the Badovci Lake, Republic of Kosovo (Figure 1). Badovci Lake is an artificial reservoir also known as Graçanica Lake located 2 km far from this small town [14] and 17.4 km from the capital city Prishtina with the coordinates 42.6234°N and 21.2412°E. The maximal length of the lake is 4.6 km, the maximal width is 0.8 km, the surface area is 1.7 km^2^, the average depth is 29 m, and the surface elevation is 655 m [15]. The Badovci Lake supplies water to a part of the Prishtina city. In the area of the lake, three regional (NNW-SSE) trending zones of mineralization are recognized within the belt: Zone I includes Artana-(Novoberdo-)Batllavë, which is marked by extensive Neogene calc-alkaline volcanics and intrusives, follows the boundary between the Kosovo sector of the Serbo-Kosovaro-Macedonian Massif; Zone II follows from the major fault that marks the eastern margin of the Miocene Prishtina basin and many volcanic mineral complexes in the north of the Republic of Kosovo; and Zone III includes the Crnac mine and extends along a number of lead-zinc occurrences in the Vardar Zone, where it is in contact with the Drenica structural block mineralogy of the area where the Badovci Lake lies, consisting of galena, sphalerite, and pyrite ores [16].

### 2.2. Sample Collection and Preparation

Samples were collected from ten different locations S1–S10 during April 2018 in the Badovci Lake. Ten samples of sediments were taken from the bottom sediments and ten samples of water from one meter above the bottom sediments (Figure 1). The samples for analyzing sediments were taken at the depth of 0 to 15 cm by approximately 200 g mass using a portable sampler (Windaus-Labortechnik GmbH & Co. KG. D-38678 Clausthal-Zellerfeld) and water samples were taken at the same locations one meter above bed sediments by the portable sampler (KLL-S Sampler by SEBA Hydrometrie GmbH & Co. KG). The standard method USEPA 2001 [17] was used for sediment collection, while for water samples, the procedures were in accordance with the method USGS 2018 [18]. The polypropylene bottles were acid-cleaned, washed with redistilled water, and filled with water. To achieve pH about 1 [19], 1 ml of nitric acid of high grade was added. The samples of sediments were taken from the center of the catcher with extra care to avoid contamination. Sediment and water samples were transported to the Central Laboratory of the Chemistry Department, Faculty of Mathematical and Natural Sciences, University of Prishtina, Kosovo. Sediment samples were dried at room temperature than in the oven at 45°C for 48 h to constant weight. After drying, the samples were crushed by using a mill and sieved through a 0.045 mm sieve (DIN 4188).

### 2.3. Sample Digestion and Metal Extraction

The chemicals used for the preparation of the solution were of analytical grade, while water was redistilled. The Teflon vessel and polypropylene containers were cleaned, soaked in 5% nitric acid for 24 h, and then rinsed with redistilled water. For metal analysis, previously, an acidified water sample was filtered through a Whatman filter paper with a 0.45 *μ*m pore size, evaporated, and then acid solution digested according to USEPA 1994 method 200.7 [20]. The analysis of total metals in the sediment samples was processed according to the instruction by USEPA 1996 method 3050b [21]. According to the study in [11], the sequential procedure was used for fractionating heavy metals. The sequential extraction procedure was divided into five operationally defined chemical fractions: F1-exchangeable: changes in water ionic composition affect sorption-desorption processes; F2-bound to carbonates: significant trace metal contents can associate with carbonates in the sediment; F3-bound to Fe and Mn oxides: these oxides are scavengers for heavy metals and thermodynamically unstable under anoxic conditions and play a role as nodules, concretions, cement between particles, or a coating on particles; F4-bound to organic matter: under oxidizing conditions in natural waters, organic matter can be degraded, leading to the release of toxic metals; and F5-residual: metals are not expected to release in the solution under the normal conditions because they are held by minerals in their crystal structure. The procedure and detailed geochemical fractionation of sediment samples can be seen in Table 1.

### 2.4. Instrumental Analysis and Quality Assurance

Heavy metal samples were analyzed by ICP-AES with a Varian 715-ES ICP optical emission spectrometer equipped with a CETAC ultrasonic nebulizer (ICP/U-5000AT+) for better sensitivity at the Institute of Chemistry, Methodius University, Skopje, North Macedonia. Preparing the calibration curve, the multielement standard was used with *R*^2^ > 0.999. The multielement standard solution 11355‐ICP, IV Merck, was used to prepare standard solutions of 1.0, 5.0, and 10.0 *μ*g/L as a tunning solution covering a wide range of masses of the elements. For each measure, a run included blank samples. The samples were analyzed in triplicate to eliminate any specific error.

### 2.5. Metal Assessment in Sediments

The data selection in the interpretation of the geochemical results plays an important role [22, 23]. Through determining and calculating the CF values, it is possible to assess the degree of contamination by toxic metals for parameters such as the PLI (pollution load index), the EF (enrichment factor), and I_geo_ (index of geoaccumulation).

### 2.6. Pollution Load Index (PLI) and Contamination Factor (CF)

According to the study in [24, 25], an integral approach can be used for calculation of the PLI of the thirteen metals and sediment quality assessment. The pollution load index (PLI) is the square root of the multiplication of the contamination factor (CF) of metals:

(1) $$\begin{array}{c} \text{PLI}={\left(\text{CF}1\;\times \text{ CF}2\;\times \text{ CF}3\;\times \cdots \times \text{CF}n\right)}^{1/n},\\ \text{CF}\left(\text{metal}\right)=\frac{\;C\left(\text{metal}\right)}{\;C\left(\text{backgound}\right)}, \end{array}$$

where CF(metal) is the ratio between the content of each metal and the background value (taken from the Dutch List) in sediment and water samples of the study area.

The contamination factor and pollution load index are also used to evaluate the status of heavy metal contamination [26, 27]. Contamination factor values were interpreted as follows: CF < 1, low pollution; 1 ≤ CF < 3, moderate pollution; 3 ≤ CF < 6, considerable pollution; and CF ≥ 6, very high pollution [26].

### 2.7. Geoacumulation Index (I_geo_)

An assessment degree of contamination by the toxic metals could be determined by the geoacumulation index (I_geo_). According to the study in [28], I_geo_ is calculated as

(2) $$\begin{array}{c} {\text{I}}_{\text{geo}}=\log\;2\left(\frac{\text{Cn}}{1.5\text{ Bn}}\right), \end{array}$$

where Cn expresses the content of the toxic metal *n*, Bn expresses background data of the toxic metal *n*, and 1.5 is a factor of possible lithological changes [9]. The I_geo_ values can be interpreted as follows: I_geo_ ≤ 0, unpolluted; 0 ≤ I_geo_ ≤ 1, unpolluted to moderately polluted; 1 ≤ I_geo_ ≤ 2, moderately polluted; 2 ≤ I_geo_ ≤ 3, moderately to heavily polluted; 3 ≤ I_geo_ ≤ 4, heavily polluted; 4 ≤ I_geo_ ≤ 5, heavily to extremely polluted; and 5 ≤ I_geo_, extremely polluted.

### 2.8. Enrichment Factor

The EF of heavy metals have been commonly used to assess anthropogenic contamination. Element Fe was chosen as the normalizing element for identifying anomalous heavy metal contributions [29]. The EF values can be calculated by using the following equation:

(3) $$\begin{array}{c} \text{EF}=\frac{\text{C}/\text{Fe}\left(\text{sample}\right)}{\text{C}/\text{Fe}\left(\text{backgound}\right)},\; \end{array}$$

where C/Fe (sample) and C/Fe (background) represent the heavy metal-to-Fe ratios in our study and in the background sample, respectively. Generally, values of the EF can be interpreted as follows: <2, minimal; 2–5, moderate; 5–20, significant; 20–40, very high; and >40, extremely high.

### 2.9. Statistical Analysis

The Minitab 16 package was used for analyzing the statistical data. The results of heavy metals in the water and sediments were calculated by using average and standard deviation. Microsoft Excel 2010 was used for other calculations.

## 3. Results and Discussion

### 3.1. Metal Concentration in Water

The results of the toxic metal concentrations in waters of the Badovci Lake are presented in Table 2. According to Table 2, it is observed that most of the metals in waters of the Badovci Lake show increased concentrations compared to the data reported by the authors in the year 2016 [14]. Al concentration has increased, whose average concentration is 0.65 mg/L, while in the year 2016, it was 0.024 mg/L. A slight deviation is observed in Ba concentration. The results show that Cu concentration has increased about ten times. The average concentration of Fe and Mn has increased to 0.99 mg/L and 0.096 mg/L, respectively, compared to results in the year 2016. The concentrations of other analyzed metals are lower than their concentrations in the year 2016. The increased concentrations of Al, Fe, and Mn have been a concern; they are higher compared to the values reported in EC 98/83 and FTRV: aluminum has increased about three times, iron four times, and manganese about once. The concentrations of As, Co, Ni, Pb, and V are <0.01 mg/L, while the concentrations of Cd and Cr are <0.001 mg/L. The lithology of the Badovci Lake is dominated by various minerals such as serpentinite, dacite, andesite, listwaenite, and phyllite [32], which may have contributed to the increase in concentrations of some metals. Also, the mineral activity which is present in this locality as in the Kishnica mine may have contributed to the increase in the concentration of these metals. Although lying in an area of similar minerality [16, 32], due to anthropogenic activity, Badovci Lake waters have been loaded with high concentration of metals than Batlava Lake waters (Table 3).

### 3.2. Total Content of Metals in Sediments

The total metal contents in sediments of the Badovci Lake are presented in Table 4. Among sampling sites, a wide range of values were obtained for the metal content. A crucial role was played by the geological structure and land runoff in the value variation of metals. As shown in Table 4, the average content of heavy metals in sediments of the Badovci Lake was much higher than Dutch Target and Intervention Values, 2000 (the New Dutch List) [20], except As, Ba, and Zn, because the site where the Badovci Lake lies is known for its geological contents of analyzed metals [16, 32]. In waters of the Badovci Lake, a considerable amount of Fe comes mostly from the geochemical rock structure.

The average concentration of heavy metals in sediments of the Badovci Lake was in the decreasing order of Fe > Al > Mn > Ni > Cr > Pb > Ba > Zn > V > Cu > As > Co > Cd. The high values of Al, Fe, and Mn were expected due to the geological composition of the region [32]. The clearer ideas on sediment contamination by heavy metals can be achieved if they are compared with the metal concentration in other lakes in the region. On the comparison of average concentration values of heavy metals Pb, V, Ni, Cu, Cr, and Co in the Badovci Lake sediment with Dutch Target and Intervention Values and with those in Lake Balaton, Lake Ohrid, Lake Skadar, Uzunçayır Dam Lake, and basins of water bodies in Silesian, as shown in Table 5, an increased content was observed in some metals such as Ni 305 mg/kg, followed by Cr 276 mg/kg, Pb 166.83 mg/kg, V 88.8 mg/kg, Cu 61 mg/kg, and Co 13 mg/kg. As, Ba, Zn, and Cd concentration values in sediments were lower than Dutch Target and Intervention Values (the New Dutch List, 2000). Average contents of the majority elements Al, Fe, and Mn exceeded the limit by FSTRVs and Lake Skadar sediment contents. As can be seen from the results, the influence of the geological structure of soil and anthropogenic activity on the content of heavy metals in sediments of the Badovci Lake is evident.

### 3.3. Chemical Fraction of Metals in Sediments

**Figure 2:**
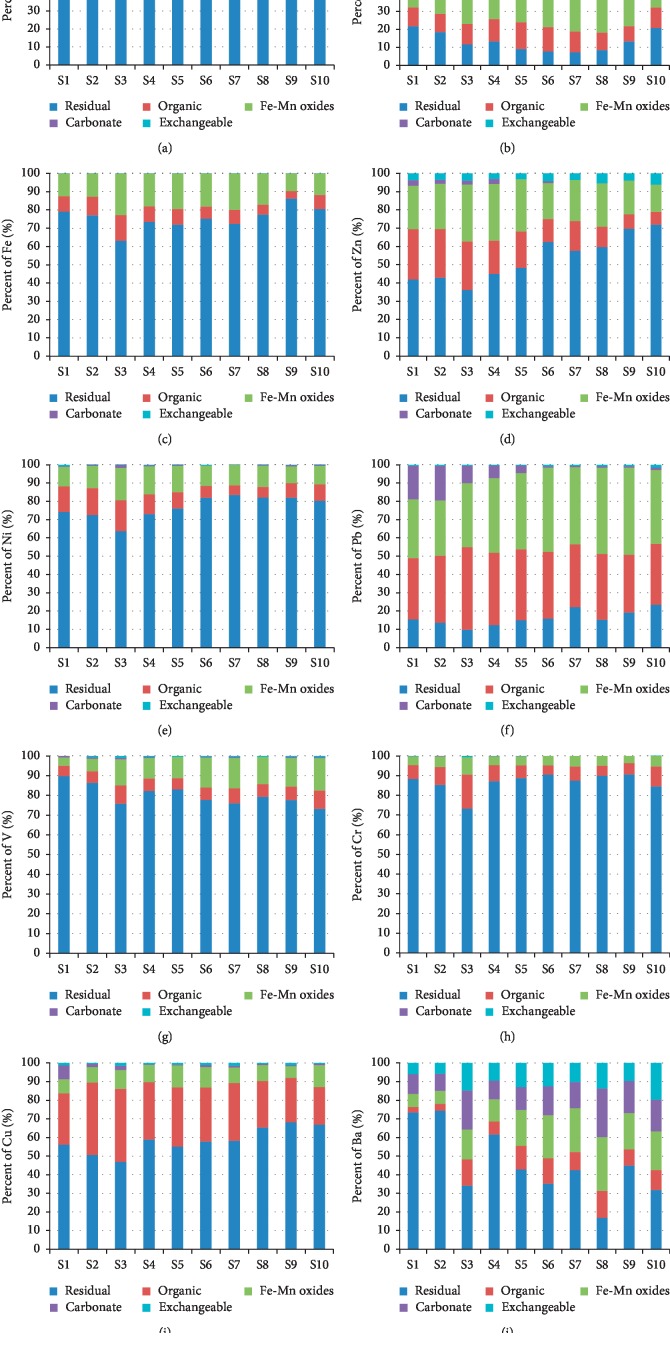
Relative distribution of metals in the sediment of the Badovci Lake from different geochemical fractions. Due to the low values of As, Co, and Cd, they are not shown (As < 10 mg/kg, Co < 10 mg/kg, and Cd < 1 mg/kg).

To obtain information about the ways and binding strength of metals associating with the sediment, the procedure of sequential extraction is proposed [24]. The percentage of metal contents derived from the sequential extraction is presented in Figure 2. According to [the study in 12], metals that are bonded to sediments in the exchangeable form are considered to be the weakest bond and equilibrate with the liquid phase becoming more easily bioavailable. Thirteen metals analyzed, which are associated with different fractions in sediments of the Badovci Lake, followed the descending order, as shown in Table 6.

In general, results of sequential extraction indicate that the residual fraction of the sediments of the Badovci Lake was dominated by Al (74–95%), Fe (63–87%), Ni (63–80%), V (75–83%), Cr (73–84%), Ba (16–74%), Zn (35–69%), and Cu (56–58%). Pb dominated in the Fe-Mn oxide fraction (22–40%) and in the organic fraction (33–39). Manganese dominated in the descending order of Fe-Mn oxide (31–48%), carbonate (19–24%), and exchangeable (3–23%) fractions (Figure 2).

These results suggested that Al, Fe, Ni, V, and Cr in the sediment of the Badovci Lake had the strongest associations with crystalline sedimentary components and were likely to reflect the geological characteristics. The concentration of Mn connected with fractions F1, F2, and F3 was dominant and under changing environmental conditions, considering the geological structure of soil [16, 32]; Mn can easily pass into water, which was proven in the water analysis. Depending on the sampling sites of collection, Mn was between 35 and 48% of the total concentration (Figure 2). Pb was present in fractions F3 and F4 more than all other fractions in the sediment. The high percentage of Pb in the organic fraction increases bioavailability. The percentage of Pb in fractions F3 and F4 was between 40 and 46% of its total concentration (Figure 2). The geological structure of the soil and mining activity have contributed to increasing the Pb content in the sediments. Cu was present, above all in fraction F3, between 20 and 40% (Figure 2). Cu has the highest affinity for bonding with organic matter. Zn associated with fraction F3 between 30 and 40% and with fraction F5 between 29 and 70% of the total concentration in the sediments (Figure 2). The Kishnica mine mainly extracts Pb and Zn minerals by the flotation method to produce Pb and Zn concentrates. This activity releases many wastes by these metals, which could be infiltrated in various ways. Ba was associated with different fractions depending on the location of the sediment in the Badovci Lake. At points S1 and S4, fraction F5, between about 70 and 75%, was dominant.

### 3.4. Assesment of Metal Pollution in Sediments

**Figure 3:**
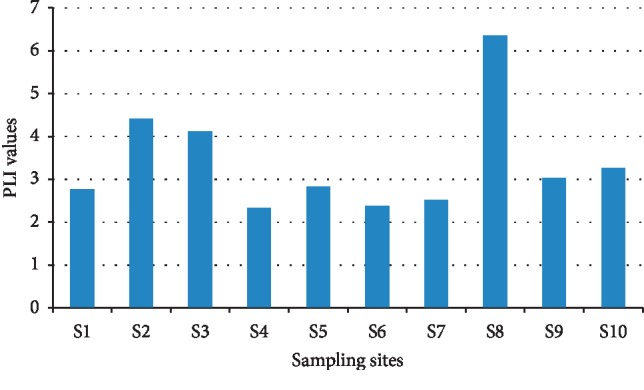
Pollution load index (PLI) values for heavy metals in sampling sites of the Badovci Lake. PLI = 0, perfection; PLI = 1, baseline level; PLI > 1, progressive deterioration [39].

**Figure 4:**
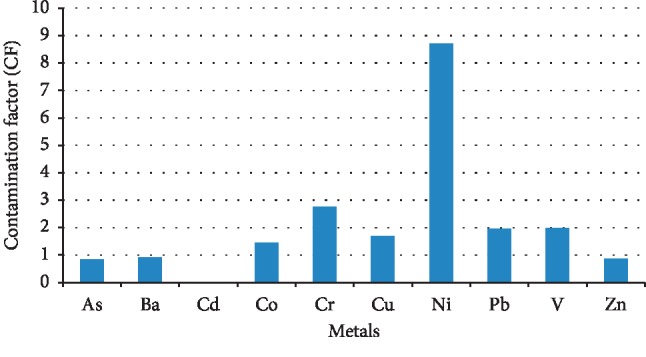
Contamination factor (CF) values for heavy metals in the sediments of the Badovci Lake. Low degree, CF < 1; moderate degree, 1 < CF < 3; considerable degree, 3 < CF < 6; very high degree, CF > 6 [22].

**Figure 5:**
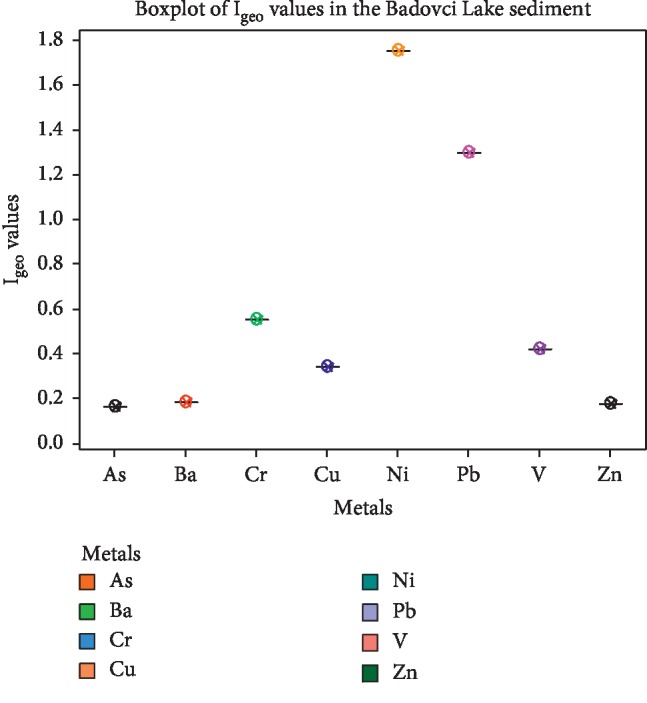
Geoacumulation index (I_geo_) values for heavy metals in the sediments of the Badovci Lake. I_geo_ ≤ 0, practically uncontaminated; 0 ≤ I_geo_ ≤ 1, uncontaminated to moderately contaminated; 1 ≤ I_geo_ ≤ 2, moderately contaminated; 2 ≤ I_geo_ ≤ 3, moderately to heavily contaminated; 3 ≤ I_geo_ ≤ 4, heavily contaminated; 4 ≤ I_geo_ ≤ 5, heavily to extremely contaminated; 5 ≤ I_geo_, extremely contaminated.

The calculated PLI values for metals in the sediment are summarized in Figure 3, which range from 2.34 to 6.35, confirming that the sediments of the studied lake were in progressive deterioration with PLI > 1. However, the higher PLI values indicated that Cr, Ni, Pb, V, and Ba are the major contributors to the sediment pollutions. The higher PLI values were shown in the sampling sites S2, S3, and S8 with PLI > 4. These high values may have come from the geological composition of the region [5] as well as the industrial activity in this area such as the Kishnica mine and its dumps. The CF values for analyzed metals showed a moderate degree of pollution with CF > 1, except for Ni which showed a very high degree of pollution (CF > 6) (Figure 4). Overall, the CF values for all metals were in the descending order of Ni > Cr > Pb > V > Cu > Co > Ba > Zn > As.  Figure 5 presents the geoaccumulation index values for the studied metals. The I_geo_ values showed the decreasing order of Ni > Pb > Cr > V > Cu > Ba > Zn > As. The sediment in the Badovci Lake indicates the uncontaminated to moderately contaminated status, whereas the I_geo_ values for Ni, Pb, and Cr indicate that the sediment is moderately contaminated. The low levels of the background sample and higher concentration levels in the analyzed sediment may result in higher values of these metals.

**Figure 6:**
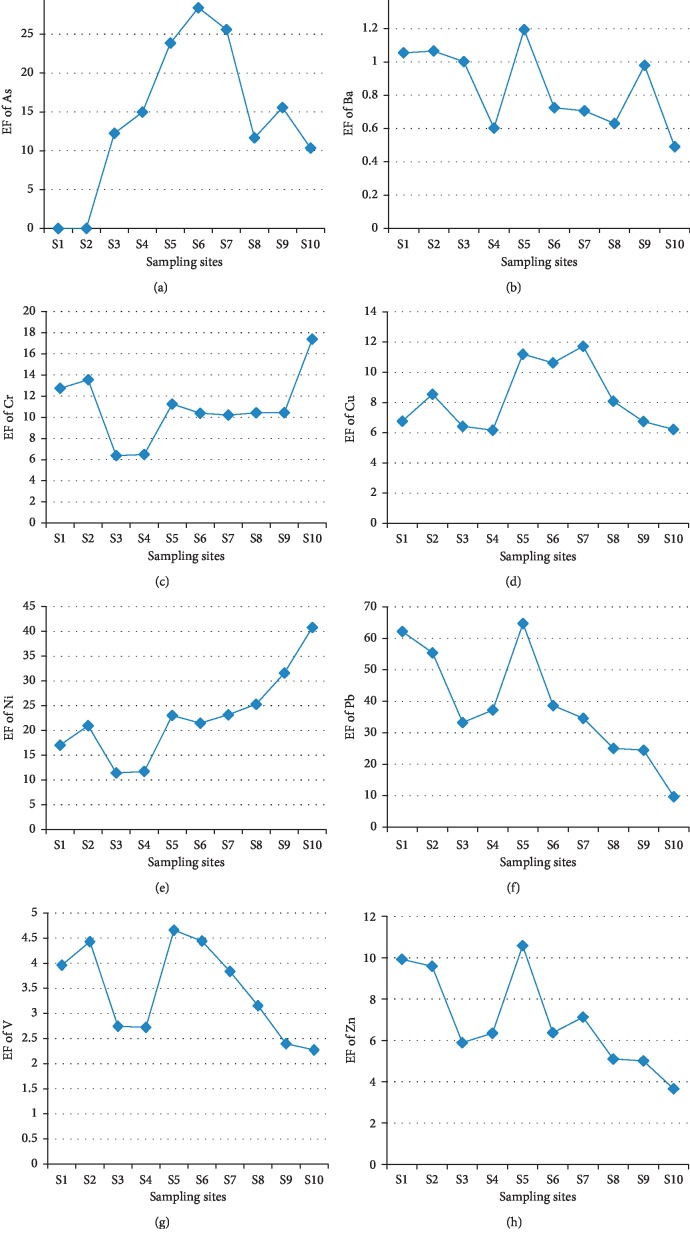
Enrichment factor (EF) values for heavy metals in the sampling sites of the Badovci Lake. <2, minimal; 2–5, moderate; 5–20, significant; 20–40, very high; >40, extremely high [3].

Figure 6 presents the spatial distributions of the calculated enrichment factor (EF) for each heavy metal studied. In general, the value of the EF for all the studied metals suggested their enrichments in sediments of the Badovci Lake. For most of the sampling sites, the EF of studied metals was higher than 2. According to the study in [3, 27], obtained results for Pb in the sediment were classified significant to very high (38.53), Ni moderate to very high (22.6), As significant (17.8), Cr moderate to significant (10.92), Cu moderate to significant (8.25), Zn moderate to significant (6.95), V moderate (3.46), and Ba minimal (0.84). In general, the Badovci Lake sediments represent significant loads for some metals such as Ni, Cr, Pb, Cu, and V. For better reflection, the obtained results were compared to those of region lakes (Table 7). Comparing the CF, EF, and I_geo_ values in Table 7 with those of the reference lakes, it is observed from the EF values that the Badovci Lake sediments are highly loaded with metals, while the CF and I_geo_ values are approximately the same.

**Figure 7:**
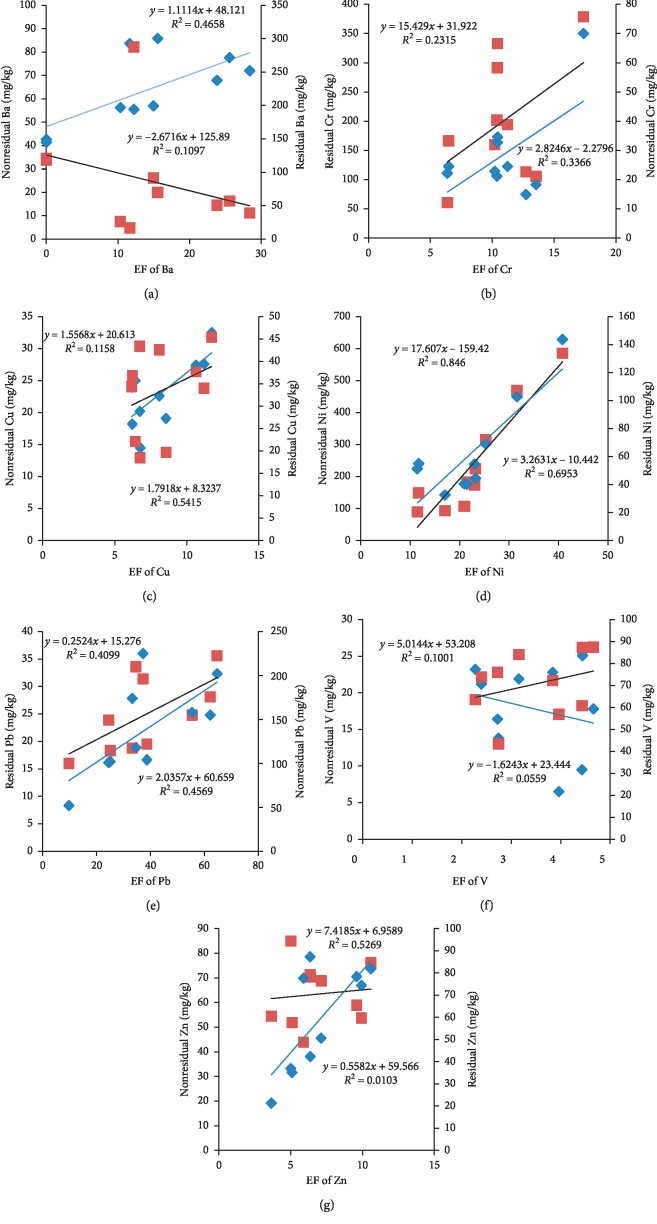
Relationship between the studied metals in the nonresidual/residual fraction and their corresponding enrichment factors (EFs). Black line, data of metals in the residual fraction and EF expressed with regression lines and equations; blue line, data of metals in the nonresidual fraction and EF expressed with regression lines and equations. Due to the low concentrations of As, Co, and Cd, they are not shown.

Due to the highest EF values and higher values of labile fractions in the sediments, attention should be paid to Pb, Ni, As, and Cr for their mobility and bioavailability in the aquatic ecosystem. According to the study in [6], concentrations of heavy metals in the residual fraction indicate lithogenic input, while the nonresidual fraction comes from anthropogenic factors.  Figure 7 represents a strong correlation between the nonresidual fraction and corresponding EF values of Ba, Cu, Ni, and Zn. The highest enrichment factor (EF) values are influenced by the anthropogenic source of heavy metals that mainly come from urbanization, industrialization, deposition of wastes, and others. Considering the lithologic composition of the soil in these parts where the lake is lying, mining activity, wastes released by mining activity, and other factors, it is possible that the high values of EF come from anthropogenic activities more than lithogenic sources.

## 4. Conclusions

Based on the obtained results from the analysis of waters of the Badovci Lake, it was found that the concentrations of Al, Fe, and Mn in the water exceed the limits set under Directive EC 98/83. Also, the comparison with research data made in the year 2016 in the same lake, as well as the comparison with the Batlava Lake or other similar lakes, shows an increase in the concentration of these elements (Al, Fe, and Mn) in the water. The sediment analysis showed that the total content of metals such as Pb, Cr, Cu, V, and Ni exceeded the limits according to the Dutch List guidelines. The average content of the total Pb was 167 mg/kg that is about twice the Dutch Target and Intervention Values, Cr 276 mg/kg twice and half, Cu 61.2 about twice, V 88.2 twice, and Ni 305 mg/kg about nine times. On the basis of the fractional analysis of metals, it was shown that Pb, Cu, and Mn were bonded to the exchangeable and organic fractions, and the possibility to pass in the water is evident in the cases of changing environmental factors. Pollution indicators such as EF, CF, and I_geo_ showed high values. Compared to the regional lakes, the EF values were much higher in the Badovci Lake, especially very high for Pb, Ni, Cu, and Cr, which indicates anthropogenic impact as a result of mining activity. It is recommended to periodically monitor the sediment and water quality.

## Figures and Tables

**Table 1:** Toxic metal fractionation in the sediments based on Tessier et al.'s [11] procedure in the present study.

Fraction	Chemical extractions	Time (h)	Temp. (°C)	Designation
F1	8 ml 1.0 M MgCl_2_, pH = 7	1	20	Exchangeable

F2	20 ml 1.0 M CH_3_COONa/CH_3_COOH, pH = 5	5	20	Bound to carbonates

F3	20 ml 0.04 M NH_2_OH·HCl/25% HAc solution	5	96 ± 3 with occasional agitation	Reducible (bound to iron and manganese oxides)

F4	5.0 ml 0.02 M HNO_3_, 5.0 ml H_2_O_2_ 30% 6.0 ml H_2_O_2_ CH_3_COONH_4_/20% (v/v) HNO_3_	2 3 0.5	85 20	Oxidizable (bound to organic matter)

F5	5 ml HNO_3_ + 10 ml HF + 10 ml HClO_4_	Until completely dissolved	Warm	Residual (bound to the soil matrix)

After each extraction stage, the samples were centrifuged and liquid-fraction-decanted, and 10 ml redistilled water was added to the residue 3 times (samples were centrifuged and water was removed) before proceeding to the next extraction.

**Table 2:** Heavy metal concentration (in mg/L) in waters of the Badovci Lake and comparison to other studies and different international guidelines.

Metals	Al	As	Ba	Cd	Co	Cr	Cu	Fe	Mn	Ni	Pb	V	Zn
DL (mg/L)	0.002	0.003	0.001	0.002	0.005	0.001	0.002	0.01	0.001	0.003	0.001	0.001	0.005
S1	0.72	<0.01	0.033	<0.001	<0.01	<0.001	0.01	1.1	0.076	<0.01	<0.01	<0.01	0.001
S2	0.44	<0.01	0.028	<0.001	<0.01	<0.001	0.033	0.67	0.053	<0.01	<0.01	<0.01	0.007
S3	0.58	<0.01	0.028	<0.001	<0.01	<0.001	0.009	0.9	0.063	<0.01	<0.01	<0.01	0.006
S4	0.41	<0.01	0.027	<0.001	<0.01	<0.001	0.017	0.58	0.051	<0.01	<0.01	<0.01	0.001
S5	0.72	<0.01	0.029	<0.001	<0.01	<0.001	0.01	1.14	0.111	<0.01	<0.01	<0.01	0.001
S6	1.43	<0.01	0.034	<0.001	<0.01	<0.001	0.009	2.29	0.285	<0.01	<0.01	<0.01	0.001
S7	1.2	<0.01	0.03	<0.001	<0.01	<0.001	0.003	1.81	0.16	<0.01	<0.01	<0.01	0.001
S8	0.23	<0.01	0.024	<0.001	<0.01	<0.001	0.001	0.3	0.042	<0.01	<0.01	<0.01	0.001
S9	0.23	<0.01	0.024	<0.001	<0.01	<0.001	0.003	0.29	0.025	<0.01	<0.01	<0.01	0.001
S10	0.55	<0.01	0.029	<0.001	<0.01	<0.001	0.211	0.82	0.09	<0.01	<0.01	<0.01	0.001
Average	0.65	<0.01	0.028	<0.001	<0.001	<0.001	0.031	0.99	0.096	<0.01	<0.01	<0.01	0.002
±SD	±0.39	—	±0.003	—	—	—	±0.064	±0.64	±0.077	—	—	<0.01	±0.002
^a^EC 98/83	0.2	0.01	—	0.005	—	0.05	2	0.2	0.05	0.02	0.01	—	—
^b^FTRV	0.087	0.15	0.004	0.002	—	0.011	0.009	—	—	0.052	0.002	<0.10	0.118
^c^Badovci Lake, 2016	0.0420	<0.010	0.0260	0.0020	—	0.0020	0.0030	0.0360	0.0039	0.0015	<0.01	0.001	0.041

DL: detection limit; ^a^EU Directive 1998/83 Drinking Water Standards (https://eur-lex.europa.eu/legal-content/EN/TXT/?uri=celex%3A31998L0083) [30]; ^b^freshwater toxicity reference value proposed by USEPA 1999 [31]; ^c^Gashi et al. [14].

**Table 3:** Comparison of metal concentration in waters (mg/L) between Badovci Lake and Batlava Lake.

		Badovci Lake, current study	Badovci Lake [14]	Batlava Lake [26]
Metals	Unit	Results/average	Results/average	Results/average
Al	mg/L	0.65	0.042	0.26
As		<0.01	<0.01	<0.01
Ba		0.028	0.026	0.047
Cd		<0.001	0.002	—
Co		<0.001	—	<0.001
Cr		<0.001	0.002	0.0016
Cu		0.031	0.003	0.0077
Fe		0.99	0.036	0.23
Mn		0.096	0.0039	0.024
Ni		<0.01	<0.01	<0.01
Pb		<0.01	<0.01	<0.01
V		<0.01	<0.01	<0.01
Zn		0.002	0.041	0.053

**Table 4:** Heavy metal content (in mg/kg dw) in the sediment of the Badovci Lake and comparison to other studies and different international guidelines.

Metals	Al	As	Ba	Cd	Co	Cr	Cu	Fe	Mn	Ni	Pb	V	Zn
DL (mg/kg)	0.01	0.01	0.01	0.1	0.1	0.05	0.01	0.005	0.005	0.01	0.01	0.01	0.01
S1	6214	<10	115	<1	<10	203	32.3	11637	268	139	183	66.4	115
S2	3865	<10	121	<1	<10	225	42.5	12119	243	178	170	77.3	116
S3	32556	21.5	232	<1	<10	216	65.1	24724	420	198	208	97.7	145
S4	32892	29.9	159	<1	<10	250	71.2	28146	557	231	265	110.4	178
S5	16803	21.2	140	<1	<10	193	57.5	12518	513	202	205	84	132
S6	15951	29.5	99.3	<1	<10	208	63.7	14620	682	220	143	93.5	93.1
S7	14995	27.4	99.8	<1	<10	211	72.5	15076	820	245	132	83.3	107
S8	9167	19.2	137	<1	<10	331	76.9	23183	1008	411	147	105.3	118
S9	20656	28.2	234	<1	<10	365	70.5	25517	1502	565	158	88.1	128
S10	16920	17.1	107	<1	13	555	59.4	23297	584	666	57.3	76.3	85.1
Average	17002	24.2	144	<1	13	276	61.2	19084	660	305	167	88.2	122
^a^T&I DL	—	29/55	160/625	0.8/12	20/240	100/380	36/190	—	—	35/210	85/530	42	140/720
^b^FSTRV	14000	6	20	0.6	—	26	16	—	—	16	31	—	110
^c^Lake Skadar	13763	—	—	0.5	—	63.5	26.8	17400	529	88.9	18.1	—	53.2

DL: detection limit; ^a^Dutch Target and Intervention Values, 2000 (the New Dutch List), Version, February 4, 2000 (http://www.esdat.net) [33]; ^b^freshwater sediment toxicity reference value proposed by USEPA 1999 [31]; ^c^Vemic et al. [29].

**Table 5:** Comparison between sediment metal contents (mg/kg) in the Badovci Lake (present study) and similar lakes within the Balkan region and other lakes.

		Badovci Lake Current study	Lake Balaton [34]	Lake Ohrid [35]	Lake Skadar [36]	Uzunçayır Dam Lake [37]	Basins of water bodies in Silesian Upland, Southern Poland [38]
Metals	Unit	Average	Min–max	Min–max	Min–max	Mean	Min–max

Al	mg/kg	17002	—	—	—	—	
As		24.2	—	—	—	9.08	5.0–7.0
Ba		144	—	—	—	—	474–530
Cd		<1	0.1–0.7	—	0.05–1.01	0.17	0.5–0.5
Co		13	1.7–17	6.07–117	—	—	2.0–6.0
Cr		276	5.7–66	2.2–576	10.28–82.60	97.25	53–72
Cu		61.2	0.7–36	1.51–34.9	7.65–28.53	29.45	8.0–20
Fe		19084	—	3510–77400	—	31740	—
Mn		660	—	—	—	631.6	—
Ni		305	4.4–5.5	10.4–1501	23.6–136.1	237.8	10–16
Pb		167	2.4–160	—	3.71–60.1	11.32	19–33
V		88.2	—	—	—	—	35–44
Zn		122	13–150	6.99–42.4	25.7–87.2	62.81	75–116

**Table 6:** Descending order of analyzed metal fractions in the sediment of the Badovci Lake.

Metals	Descending order of metals by the chemical fraction
Fe	Residual > Fe-Mn oxides > organic > carbonate > exchangeable
Al	Residual > organic > Fe-Mn oxides > carbonate > exchangeable
Mn	Fe-Mn oxides > carbonate > exchangeable > organic > residual
Ba	Residual > Fe-Mn oxides > carbonate > exchangeable > organic
Zn	Residual > Fe-Mn oxides > organic > exchangeable > carbonate
V	Residual > Fe-Mn oxides > organic > carbonate > exchangeable
Cr	Residual > organic > Fe-Mn oxides > carbonate > exchangeable
Ni	Residual > Fe-Mn oxides > organic > carbonate > exchangeable
Pb	Fe-Mn oxides > organic > residual > carbonate > exchangeable
Cu	Residual > organic > Fe-Mn oxides > carbonate > exchangeable
As	Residual > organic = Fe-Mn oxides = carbonate = exchangeable
Cd	All fractions are <1 mg/kg
Co	All fractions are <1 mg/kg

**Table 7:** Comparison of the pollution indicators (CF, EF, and I_geo_) between Badovci Lake and other lakes.

	Contamination factor				Enrichment factor				Index of geoaccumulation			
	Badovci Lake	Viroi Lake [40]	Uzunçayır Dam Lake [37]	Silesian basin [38]	Badovci Lake	Viroi Lake [40]	Uzunçayır Dam Lake [6]	Silesian basin [38]	Badovci Lake	Viroi Lake [40]	Uzunçayır Dam Lake [37]	Silesian basin [38]
As	0.83	—	5.04	—	17.83	—	0	15.00	0.167	—	—	0.0–5.20
Ba	0.90	—	—	—	0.844	—	—	1.08	0.181	—	—	1.55–3.72
Cd	0	—	1.09	—	0	—	1.61	109.8	0	—	—	1.77–6.89
Co	1.44	—	—	—	0	—	—	8.25	0	—	—	0.74–3.91
Cr	2.75	—	1.08	—	10.92	1.016	1.61	2.11	0.55	0.56	—	2.35–3.91
Cu	1.69	—	0.65	—	8.25	2.15	0.96	10.64	0.34	0.73	—	0.0–3.73
Ni	8.71	—	3.49	—	22.64	4.51	5.27	4.75	1.75	1.59	—	0.28–3.17
Pb	1.92	—	0.56	—	38.53	3.5	0.83	87.22	1.3	1.22	—	0.55–5.18
V	1.98	—	—	—	3.46	—	—	2.08	0.424	—	—	1.90–2.96
Zn	0.87	—	0.66	—	6.95	—	0.97	47.04	0.177	—	—	2.60–6.63

## Data Availability

The data used to support the findings of this study are available from the corresponding author upon request.
